# Molecular Interactions between APIs and Enteric Polymeric Excipients in Solid Dispersion: Insights from Molecular Simulations and Experiments

**DOI:** 10.3390/pharmaceutics15041164

**Published:** 2023-04-06

**Authors:** Krishna M. Gupta, Xavier Chin, Parijat Kanaujia

**Affiliations:** 1 Institute of Sustainability for Chemicals, Energy and Environment (ISCE2), Agency for Science, Technology and Research (A*STAR), 1 Pesek Road, Jurong Island, Singapore 627833, Singapore; 2 Department of Pharmacy, National University of Singapore, 18 Science Drive 4, Singapore 117559, Singapore

**Keywords:** molecular dynamics simulation, interaction energy, hydrogen bonding, solid dispersion, hot melt extrusion, amorphous formulation

## Abstract

Solid dispersion of poorly soluble APIs is known to be a promising strategy to improve dissolution and oral bioavailability. To facilitate the development and commercialization of a successful solid dispersion formulation, understanding of intermolecular interactions between APIs and polymeric carriers is essential. In this work, first, we assessed the molecular interactions between various delayed-release APIs and polymeric excipients using molecular dynamics (MD) simulations, and then we formulated API solid dispersions using a hot melt extrusion (HME) technique. To assess the potential API–polymer pairs, three quantities were evaluated: (a) interaction energy between API and polymer [electrostatic (E_coul_), Lenard-Jones (E_LJ_), and total (E_total_)], (b) energy ratio (API–polymer/API–API), and (c) hydrogen bonding between API and polymer. The E_total_ quantities corresponding to the best pairs: NPX-Eudragit L100, NaDLO–HPMC(P), DMF–HPMC(AS) and OPZ–HPMC(AS) were −143.38, −348.04, −110.42, and −269.43 kJ/mol, respectively. Using a HME experimental technique, few API–polymer pairs were successfully extruded. These extruded solid forms did not release APIs in a simulated gastric fluid (SGF) pH 1.2 environment but released them in a simulated intestinal fluid (SIF) pH 6.8 environment. The study demonstrates the compatibility between APIs and excipients, and finally suggests a potential polymeric excipient for each delayed-release API, which could facilitate the development of the solid dispersion of poorly soluble APIs for dissolution and bioavailability enhancement.

## 1. Introduction

One of the most important challenges in the delivery of active pharmaceutical ingredients (APIs) faced by formulation scientists is how to counter the low aqueous solubility of APIs [1,2,3]. Amorphous solid dispersion formulations of APIs with excipients is considered to be a promising strategy to improve the oral bioavailability of poorly soluble APIs [4]. Consequently, solid dispersions of APIs in excipients (water-soluble polymeric carriers), which enhance aqueous solubility over the crystalline counterpart, have been widely applied in pharmaceutical formulations. Molecules in amorphous solids exist at higher energy than those in a crystalline state; therefore, the energy penalty required to dissociate these molecules is lower, resulting in a higher solubility than a crystalline form [5].

To develop the amorphous solid dispersion of APIs, a hot melt extrusion (HME) technique has emerged as a potent processing technology and several commercial HME products are available on the market or are under late-stage development [6,7]. In HME, a mixture of drug, excipient and plasticizer, if required, is heated at high temperatures (below the melting point of API) and intensively mixed using a twin-screw extruder to yield a homogeneous product. On the other hand, when using HME, an API is dispersed into a polymer matrix to produce solid dispersions with improved bioavailability of poorly soluble drugs [8,9]. The other method of solid dispersion preparation is the common solvent method, in which both drug and carrier are dissolved in a common solvent and then the solvent is evaporated by spray drying [10] or freeze drying to obtain the solid dispersion product [11]. Compared to the traditional milling or solvent-based methods, HME technology has received significant attention for solid dispersion formulations owing to various advantages such as fewer processing steps, decreased processing time, continuous operation, solvent-free operation, superior mixing capabilities, and potential for automation [12,13]. Products developed using a hot melt extrusion process have been approved by regulatory agencies worldwide for human use [6]. Some of the marketed products include Kaletra^®^, Norvir^®^, FenoglideTM, Verapamil and Posaconazole.

A major challenge for the development of amorphous solid dispersion formulations occurs due to the amorphous nature of APIs as they are thermodynamically unstable and tend to recrystallize [14]. To mitigate this issue, polymeric additives are usually added as excipients in the formulation; these additives prevent the recrystallization process and hence improve physical stability [15]. A wide range of polymeric excipients have been commercially utilized including, but not limited to, hydroxypropyl methylcellulose (HPMC), hydroxypropyl methylcellulose phthalate [HPMC(P)], hydroxypropyl methylcellulose acetate succinate [HPMC(AS)], hydroxypropyl cellulose (HPC), polyvinylpyrrolidone (PVP), polyvinylpyrrolidone-vinyl acetate copolymer (PVP-VA), and polyethylene glycol (PEG) [16]. Among the employed excipients, cellulosic polymers are known to be superior in the inhibition of API crystallization [17,18]. Owing to three substitution positions on each D-glucose monomeric unit of cellulose polymer, a large degree of freedom exists regarding the design of new candidates in terms of both the substitution patterns and the degree of substitutions. Nonetheless, this presents a technological challenge in designing a polymer with optimal properties for a given API [19].

For the successful development of a solid dispersion formulation, understanding of the intermolecular interactions between APIs and polymeric carriers is crucial [20,21]. In particular, molecular interactions between various API molecules and polymeric excipients correlating their compatibility/miscibility are essential for the rational design and screening of formulation systems [19,22,23,24,25]. From this perspective, Meng et al. highlighted the role of molecular interactions between a poorly soluble drug (curcumin) and various hydrophilic polymers such as PVP, Eudragit EPO (EPO), HPMC and PEG in the successful formulation of solid dispersion using Fourier transform infrared and Raman spectroscopy [26]. Various experimental methods such as glass transition temperature (Tg), Raman mapping, X-ray diffraction data, solid state nuclear magnetic resonance (NMR) spectroscopy, and atomic force microscopy (AFM) have been used to qualitatively examine drug–polymer miscibility [27]. Recently, Lu et al. have investigated molecular interaction between posaconazole and HPMCAS polymer in amorphous solid dispersions using 19F magic angle spinning (MAS) nuclear magnetic resonance (NMR) techniques [28].

In addition to experimental explorations, molecular dynamics (MD) simulation has been widely employed as a promising tool to determine API-polymer miscibility in solid dispersions as well as formulation design [29,30,31]. For instance, Yani et al. have performed MD simulations to predict the miscibility of API in various ionic and non-ionic polymeric excipients for solid dispersion systems by evaluating Hansen’s solubility parameter, hydrogen-bonding interaction energy and hydrogen-bond lifetime analysis [25]. By combining experimental and MD simulation techniques, Gong and co-workers have investigated the state evolution of norfloxacin (NFX) in solid dispersions with three commonly used excipients, namely, PVP, HPMC, and HPMC(P). It was demonstrated that conversion from an amorphous NFX to a hydrated state is possibly due to the dominating self-protonation of NFX over other interactions (NFX−polymer hydrogen bonding or ionic interaction); however, enhanced NFX−NFX aggregation leads to conversion to an anhydrous crystalline state [32]. Very recently, by integrating experimental and MD simulation approaches, Kabedev et al. evaluated the underlying mechanism of β-lactoglobulin stability in solid dispersions of indomethacin [33]. Although the pace of growth in the understanding of drug−excipient interaction towards stabilizing amorphous solid dispersions of poorly water-soluble drugs has been rather encouraging, understanding of molecular interactions between delayed-release APIs and polymers governing API formulation and dissolution is largely elusive and hinders the development of solid dispersion in pharmaceutical applications.

Our work involved using both MD simulation and experimental techniques to gain a better understanding of how delayed-release drug APIs interact with enteric polymeric excipients at the molecular level. This knowledge can help speed up the development of solid dispersions for delayed-release applications. It is worthwhile to note that the selected APIs are available in enteric-coated dosage form on the market; therefore, they are the ideal candidates for solid dispersion formulations. Precisely, first the compatibility between APIs and polymeric excipients was investigated by accessing molecular interactions using molecular dynamics (MD) simulations, which were later used to identify the potential API-polymeric pairs for solid dispersions. Then, solid dispersions of the suggested API-excipient pairs were prepared using HME to gauge the feasibility of the pairs, followed by release studies in both simulated gastric fluid (SGF) with a pH of 1.2 and simulated intestinal fluid (SIF) with a pH of 6.8.

## 2. Materials and Methods

### 2.1. Simulation Models and Methods

**Figure 1:**
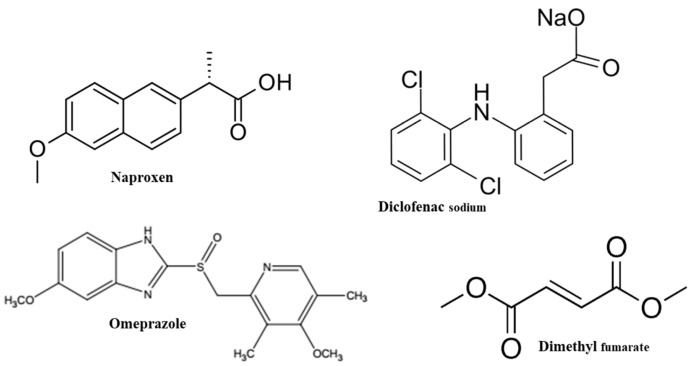
2D structures of APIs.

**Figure 2:**
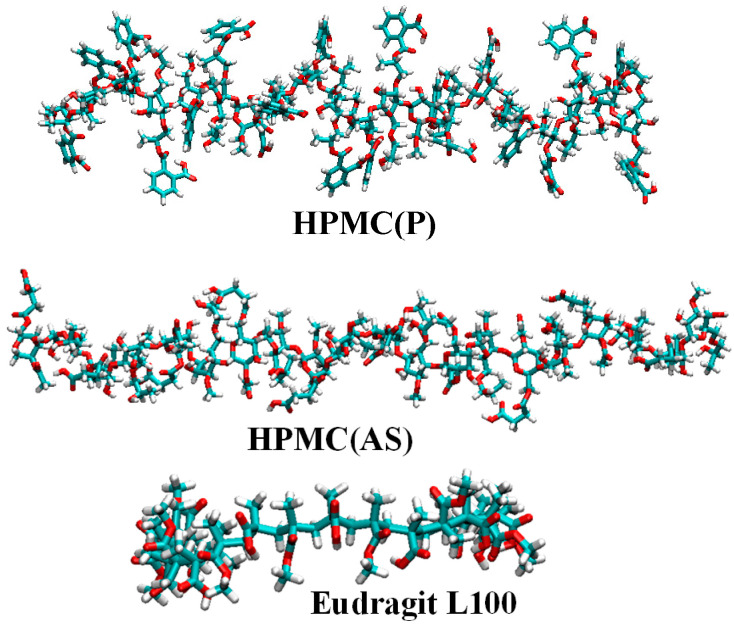
Atomic structures of polymer chains. Color code: O, red; N, blue; C, cyan; H, white.

For each API, first, MD simulations were carried out to identify the potential polymeric excipients suitable for pairing. To do so, three commonly used polymeric excipients, namely, hydroxypropyl methylcellulose phthalate [HPMC(P)], hydroxypropyl methylcellulose acetate succinate [HPMC(AS)], and Eudragit L100 were chosen. Figure 1 depicts the molecular structures of drug APIs, namely, naproxen (NPX), diclofenac sodium (NaDLO), dimethyl fumarate (DMF), and omeprazole (OPZ). For each polymer, Figure 2 represents the molecular structure of a polymeric chain with 10 monomer units. Similar to our previous work [34], we first constructed a repeat unit of each polymer, and then created a polymer chain with 10 monomer units using the *Polymer Builder* module in Materials Studio [35]. These polymer chains were geometrically optimized using the *forcite* module in Materials Studio. After optimizing the structures, input parameter files were generated for the MD simulations. The optimized potentials for liquid simulations all-atom (OPLS-AA) force field was used to describe the parameters for both APIs and polymeric structures [36]. Using the MKTOP tool, parameter files were created for most structures, and the TPP-MKTOP tool was used for a few structures [37,38]. The atomic charges were adjusted based on the OPLS-AA force field. The Lennard-Jones (LJ) and Coulombic (Coul.) potentials were used to describe the non-bonded interactions.

**Figure 3:**
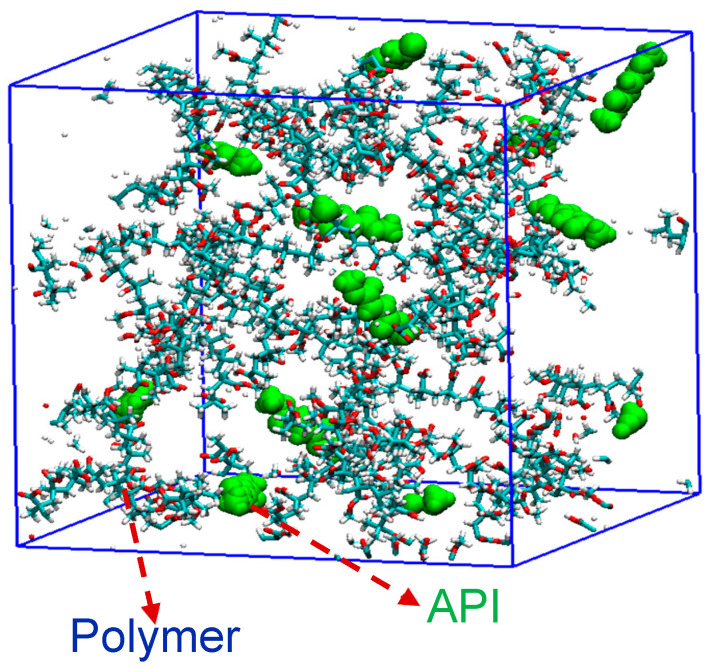
A representative simulation snapshot for the mixture of API molecules and polymeric excipient. The API molecules are shown in green.

MD simulations were conducted in four separate sets. In the first set, the crystal structure of each drug API (NPX or NaDLO or DMF or OPZ) was simulated for 5 ns using isothermal and isobaric (NPT) MD simulation. This set of simulations was performed to verify the force field used herein. In the second set, a cubic simulation box (~6 nm each side) was built for each polymer excipient [HPMC(P) or HPMC(AS) or Eudragit L100]. Then, 10 API molecules of each drug were ramdomly added into the simulation boxes, resulting in 12 different simulation systems. The number of polymer chains was added in such a way that all systems maintained 5 wt% API and 95 wt% polymer excipients. For each system, 10 ns isothermal and isochoric (NVT) MD simulations were performed at 300 K, and the last 8 ns trajectory was used for analysis. A representative simulation snapshot for this type of system is shown in Figure 3. The 2nd set was aimed at screening and identifying a suitable polymeric excipient for each drug API.

Experiments were conducted to assess the feasibility of mixing the API and polymeric excipient in a solid state once appropriate pairs of drug APIs and polymeric excipients were identified through MD simulations. The experimental procedures are elaborated later in this section. Afterwards, simulation systems were built for feasible API and excipient pairs in the third set, with a different API loading of 50 wt%. Similarly to the 2nd set, for this set, each system was simulated for 10 ns NVT MD simulations. The aim of this set was to analyze the effect of loading. In the final set, the simulations were performed at various temperatures.

The GROMACS v5.1.2 package was used to conduct all simulations [39]. The first step in all simulations was energy minimization using the steepest descent with a truncation force of 1000 kJ/mol.nm, followed by MD simulations. The Maxwell–Boltzmann distribution was used to generate initial velocities for the MD simulations. The velocity-rescale thermostat with a relaxation time of 0.1 ps was applied to control the temperature of the simulation systems. The particle-mesh Ewald summation method was adopted to calculate Coul interactions with a grid spacing of 1.2 Å. The LJ interactions were calculated using a cutoff of 14 Å. The leap-frog algorithm with a time step of 2 fs was used to integrate the equations of motion. In all three dimensions, periodic boundary conditions were enforced. Simulation snapshots were created in VMD (version 1.9.3) software using simulation trajectories [40].

### 2.2. Experimental Description

Following the simulation, API polymer mixtures corresponding to 5 wt% and 50 wt% were prepared by accurately weighing powdered API and polymers in a screw-cap bottle and mixing them homogenously using Powder mixer (Alphie, 2.5L 3D Powder Mixer, Hexagon Products, Por, Guj, India) at 50 RPM for 30 min. NaDLO and OPZ were purchased from Jai Radhe Sales India. DMF and NPX base were supplied by Sigma-Aldrich, St. Louis, MO, USA. HPMC AS (LF grade) and HPMC-P (55 grade) was kindly provided by Shine Etsu Chemical Co. (Tokyo, Japan). Eudragit L100-55 was provided by Evonik Industries (Darmstadt, Germany). High-performance liquid chromatography (HPLC) grade solvents were supplied by Fischer Scientific Pte. Ltd., (Pandan Cres., Singapore) and other reagents were supplied by Sigma, St. Louis, MO, USA and used as supplied. The solubilities, melting points, and degradation temperatures of the API and polymers are shown in Table S1. The API polymer physical mixture was fed manually to a preheated co-rotating twin-screw hot melt extruder (Prism Eurolab 16 Melt extruder from Thermo Scientific, Karlsruhe, Germany) rotating at 100 RPM. The temperature profile and screw speed were set to ensure transport, melting and mixing at the respective zones on the screw. The temperatures used in the different zones are shown below in Table 1.

For extruding solid dispersions in the form of cylindrical strands, a rod die with a 2 mm orifice was employed. The extruded strands were gathered on a conveyor belt, cooled with air, and then kept in screw-capped glass bottles under low humidity conditions (25% relative humidity) at room temperature. The resulting extrudates were subjected to ball milling using a stainless-steel vessel with a 1.5-cm diameter stainless-steel ball, at a frequency of 30 Hz for 2 min, using the MM 200 Retsch GmbH equipment from Haan, Germany. The milled extrudates were utilized for analyzing and characterizing the solid dispersions.

#### 2.2.1. Powder X-ray Diffraction Analysis

A Bruker D8 Advance powder X-ray diffractometer, which utilizes Cu Kα radiation (λ = 1.540 60 Å), an acceleration voltage of 35 kV, and a current of 40 mA power, was used to collect Powder X-ray diffraction (PXRD) data. Samples were scanned using a continuous scanning mode in the 2θ range from 5° to 50°, with a scan rate of 5° min^−1^.

#### 2.2.2. FTIR Spectroscopy

A Frontier FT-IR/NIR Spectrometer (PIKE Technologies I, PerkinElmer) equipped with a mid-infrared (MIR) triglycine sulfate (TGS) detector was used to obtain FTIR transmission spectra of the APIs, physical mixtures, and extruded samples. A small quantity of powdered sample was secured on the sample holder and scanned over a range of 4000–650 cm^−1^ at a scan speed of 0.2 cm^−1^S^−1^ with a spectral resolution of 4 cm^−1^.

#### 2.2.3. Dissolution Study

The extruded samples were broken as granules and passed through sieve no. 18 and retained in sieve no. 30 for dissolution studies. USP type 2 dissolution apparatus (Agilent Technologies) with baskets was employed for this study. All the samples were tested using the following sequence:

(1) In Simulated Gastric fluid (SGF) pH 1.2 for 2 h: The samples were placed in a basket and dilution was tested for 2 h in SGF pH 1.2 with sample collections at 15, 30, 60, 90 and 120 min. A 2 mL sample was withdrawn after each time interval and fresh media were replenished. The samples were filtered through a 0.45 µm syringe filter and analyzed by HPLC.
(2) In Simulated Intestinal fluid (SIF) pH 6.5 for 2 h: After 2 h in SGF, the dissolution medium was changed to SIF and the basket was lowered to run the same sample again. Samples were collected at 5, 10, 15, 30, 60, 90 and 120 min and 2 mL of fresh SIF was replenished after each collection. The samples were filtered through a 0.45 µm syringe filter and analyzed by HPLC.

For NaDLO solid dispersion dissolution, granules equivalent to 50 mg of NaDLO were used, whereas for DMF solid dispersion dissolution, granules equivalent to 120 mg DMF were used.

## 3. Results and Discussion

### 3.1. Force Field Validation of Drug API

To validate the force field of API molecules, first, the unit cell of each drug crystal was downloaded from the Cambridge Crystallographic Data Centre (CCDC) database. Then, the unit cell of each drug was extended in all directions to ensure that the dimensions in each direction were either close to or higher than 50 Å. To do so, NPX, NaDLO, DMF, and OPZ were extended to 4 × 9 × 7, 5 × 2 × 5, 14 × 10 × 7, and 5 × 5 × 5, respectively. After MD simulation, from the first set of simulations, densities and lattice parameters of all crystals were estimated and compared with the literature, as tabulated in Table 2. For all crystal structures, the simulated densities and lattice parameters show <3% deviation from the experimental values, which reflects the reliability of the force field adopted for the simulation.

### 3.2. Evaluation of Drug-Polymer Pairs for Solid Dispersion Formulations

**Figure 4:**
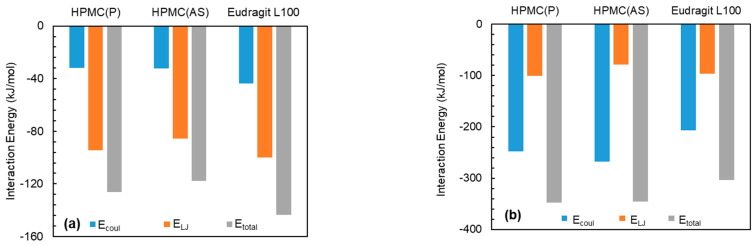

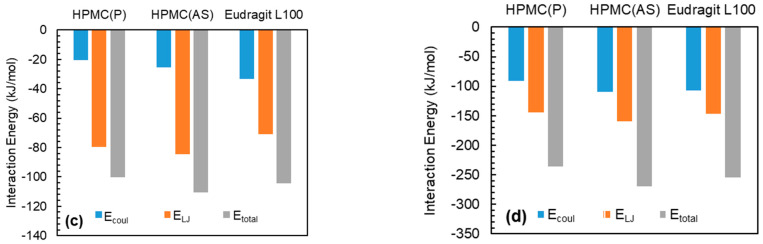
Interaction energies in terms of electrostatic (E_coul_), Lenard–Jones (E_LJ_), and total (E_total_) between API molecules and polymer excipients for (**a**) NPX, (**b**) NaDLO, (**c**) DMF, and (**d**) OPZ.

To identify a suitable polymeric excipient for a stabilized amorphous API in solid dispersion formulation, solubility measurement is crucial. Typically, the solubility of a molecule in a specific medium can be gauged based on the interaction energy between interacting molecules; in general, the higher the interaction energy (stronger interaction), the higher the solubility [45,46,47]. Recently, we estimated the interaction energies and energy ratios between actives and lipid excipients to accurately predict the active encapsulation tendency in excipients, while identifying the best active–excipient pairs for nanoparticle formulations [48]. Similarly, to determine the most suitable polymer excipient for each API, the study employed MD simulation trajectory to estimate interaction energies and energy ratios. A negative energy value typically indicates an attractive interaction, with a higher absolute value indicating a stronger interaction and greater compatibility. Conversely, a positive value indicates a repulsive or unfavorable interaction. Figure 4 illustrates the estimated interaction energies, including electrostatic (E_coul_), Lenard-Jones (E_LJ_), and total (E_total_), between drug API molecules and polymer excipients, which were determined in the second set of simulations.

The interaction energies between NPX and polymeric excipients were negative and were found to increase in the order of HPMC(AS) < HPMC(P) < Eudragit L100 (Figure 4a). This indicates that NPX exhibits favourable interactions with all polymeric excipients. Among the tested excipients, Eudragit L100 showed the highest interaction. The highest interaction between NPX and Eudragit L100 might be due to the presence of similar functional groups (-OCH3 and -COOH) in both (see Figure 1 and Figure 2). Owing to the strongest interaction of NPX, Eudragit L100 was expected to show higher solubility. It is worthwhile to note that the E_total_ between NPX and polymeric excipient is dominated by van der Waals interactions (E_LJ_). Similarly to NPX, NaDLO–excipient interactions were negative (Figure 4b). The E_coul_, E_LJ_, and E_total_ increased in the order of Eudragit L100 < HPMC(AS) < HPMC(P). The E_total_ between NaDLO and HPMC(P) was the highest, and thus is expected to be a potential choice. The possible reason for the highest interaction between NaDLO and HPMC(P) is the favourable interaction between hexagonal aromatic rings that are present in both structures (see Figure 1 and Figure 2). Owing to the strongest interaction of NaDLO, HPMC(P) was expected to show higher solubility. Interestingly, in contrast to NPX, the E_total_ between NaDLO and the polymeric excipient was dominated by electrostatic (E_coul_) interactions due to the ionic nature of NaDLO.

**Figure 5:**
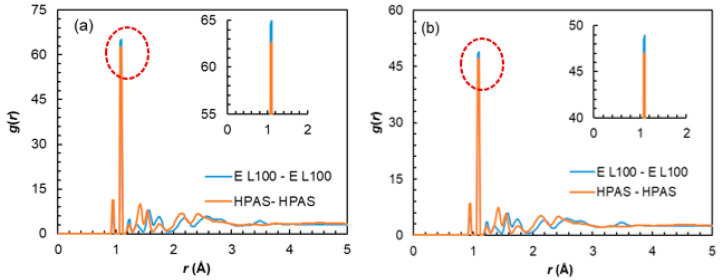
Radial distribution functions, *g*(*r*) of Eudragit L100 around Eudragit L100 and HPMC(AS) around HPMC(AS) in a mixture of these excipients with (**a**) DMF and (**b**) OPZ. The E L100 and HPAS in the figure legend indicate Eudragit L100 and HPMC(AS), respectively. The insets provide a zoomed-in view of the red circled portion.

Similarly to NPX and NaDLO, DMF–excipient and OPZ–excipient interactions were negative (Figure 4c,d), indicating favorable interactions among them. For DMF, the E_coul_, E_LJ_, and E_total_ increased in the order of Eudragit L100 < HPMC(P) < HPMC(AS), whereas for OPZ, these quantities increased in the order of HPMC(P) < Eudragit L100 < HPMC(AS). By carefully observing the chemical structures, one can say that, with a linear chain in Eudragit L100, a higher interaction with DMF (also linear chain) is expected. Interestingly, this was not observed here, instead HPMC(AS) showed the highest interaction with both DMF and OPZ. To unveil this behaviour, we further evaluated the intra-atomic Eudragit L100-Eudragit L100 and HPMC(AS)–HPMC(AS) interactions by radial distribution function *g*(*r*)as

(1) $$g_{ij}(r)=\frac{N_{ij}(r,r+\Delta r)\;V}{4\pi r^{2}\Delta r\;N_{i}N_{j}}$$

where *r* is the distance between atoms *i* and *j*, *N_i_* and *N_j_* are the numbers of atoms *i* and *j*, $N_{ij}(r,r+\Delta r)$ is the number of atoms *j* around *i* within a shell from *r* to *r* + Δ*r*, respectively. Figure 5 shows the *g*(*r*) of Eudragit L100 around Eudragit L100 and HPMC(AS) around HPMC(AS) in a mixture of these excipients with DMF and OPZ, based on all atoms. For DMF, as shown in Figure 5a, two prominent peaks are observed at *r* ~ 0.96 and 1.1 Å, indicating strong interaction between Eudragit L100 and Eudragit L100 as well as HPMC(AS) and HPMC(AS). However, Eudragit L100-Eudragit L100 interaction was stronger than HPMC(AS)-HPMC(AS), as reflected by a greater peak height in the former. Thus, owing to weaker intra-atomic HPMC(AS)–HPMC(AS) interaction, DMF is more accessible to HPMC(AS) compared to Eudragit L100 and thus has the highest interaction. Similarly, for OPZ (Figure 5b), two prominent peaks are observed at *r* ~ 0.94 and 1.08 Å, indicating strong interaction between Eudragit L100 and Eudragit L100 as well as HPMC(AS) and HPMC(AS). Due to weaker intra-atomic HPMC(AS)-HPMC(AS) interaction, OPZ is more accessible to HPMC(AS) compared to Eudragit L100, and thus shows the highest interaction. In brief, the potential choice for both DMF and OPZ is HPMC(AS). Because both DMF and OPZ are neutral molecules, as is NPX, the E_total_ between DMF/OPZ and the polymeric excipient is dominated by the van der Waals interaction rather than the electrostatic interaction.

**Figure 6:**
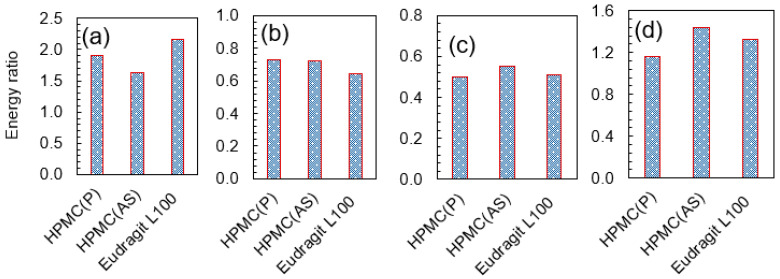
The energy ratios (**a**) NPX–polymer/NPX–NPX, (**b**) NaDLO–polymer/NaDLO–NaDLO, (**c**) DMF–polymer/DMF–DMF, and (**d**) OPZ–polymer/OPZ–OPZ.

To better understand the relative interactions between drug API and polymer excipients, energy ratios (API–polymer/API–API) were estimated for all 12 systems (Figure 6). The energy ratios lie between 0 and 2.2 depending on the system. For NPX, the energy ratios increased in the order of HPMC(AS) < HPMC(P) < Eudragit L100. For NaDLO, the energy ratios increased in the order of Eudragit L100 < HPMC(AS) < HPMC(P). For DMF, the energy ratios increased in the order of HPMC(P) < Eudragit L100 < HPMC(AS), whereas for OPZ, these quantities increased in the order of HPMC(P) < Eudragit L100 < HPMC(AS). Consistent with the API–polymer interactions prediction, the energy ratios indicate that the best pairs among the examined combinations are NPX–Eudragit L100, NaDLO–HPMC(P), DMF–HPMC(AS), and OPZ–HPMC(AS). Further, we also estimated hydrogen bonds between API and polymer excipients as they play an important role in the solubility analysis [49]. Two geometrical criteria were implemented to calculate the hydrogen bonds: (1) the distance (*r*) between a donor and an acceptor ≤ 3.5 Å and (2) the angle of hydrogen-donor-acceptor, *α* ≤ 30° [50]. Table S2 represents the number of hydrogen bonds between APIs and polymers per API on a molecular basis. All the APIs were observed to form hydrogen bonds with polymer excipients. Being ionic in nature, NaDLO showed a greater number of hydrogen bonds compared to the other APIs. Among non-ionic APIs, OPZ had a higher number of hydrogen bonds due to the presence of more oxygen atoms. To visualize the interaction patterns between APIs and polymers corresponding to the best pairs, Figure S1 shows MD simulation snapshots in NPX–Eudragit L100, NaDLO–HPMC(P), DMF–HPMC(AS), and OPZ–HPMC(AS). Following this, experiments were performed to analyze the feasibility of the suggested API-excipient pairs by simulations. Particularly, HME experiments were performed to prepare the solid dispersions of the recommended pairs, followed by dissolution experiments in an actual interstitial environment.

### 3.3. Evaluation of Drug-Polymer Pairs by Experiment

#### 3.3.1. Solid Dispersion by Hot Melt Extruder Experiments

The selected API polymer physical mixtures were melt-extruded under the conditions given in the Table 1. Extrudates of NaDLO and DMF containing 5% *w*/*w* API were glassy and transparent, whereas extrudates containing 50% *w*/*w* API were opaque. The charring of API was observed during the extrusion of NPX with Eudragit L-100. The degradation temperature of NPX has been reported to be 196 °C [51], which is higher than the extrusion temperature required to extrude Eudragit L100. The NPX was successfully extruded with Eudragit L100 by adding 10% triethyl citrate as a plasticizer to lower the extrusion temperature [52]. The OPZ degraded and formed a black product when extruded with HPMC-AS. Therefore, it was concluded that, among the suggested pairs, NaDLO–HPMC(P) and DMF–HPMC(AS) were successfully prepared. OPZ is an acid liable drug, and it is reported to degrade in the presence of enteric polymer in solution [53] and even in a solid state [54]. Sharma et al. studied the solid–state interactions between OPZ and various enteric polymers at the core–coat interface. In the acidic medium (including in the presence of enteric polymers), OPZ molecules rearrange and form a pyridinium salt, which binds selectively and irreversibly with the proton pump H+/K+-ATPase at the parietal cell secretory membrane [55]. To protect OPZ molecules from the enteric polymer, a sub-coating of neutral polymer such as HPMC and amylopectin was applied, which acts as barrier between OPZ and enteric polymer [56].

#### 3.3.2. PXRD and FTIR

**Figure 7:**
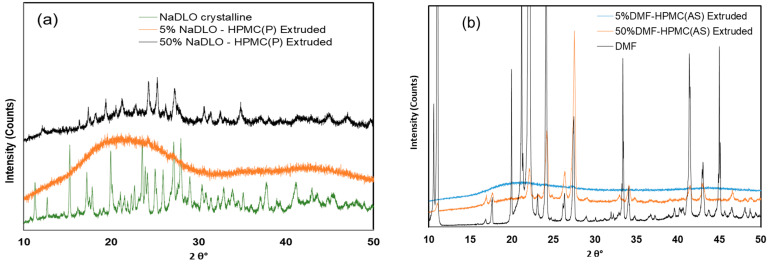
PXRD pattern of (**a**) NaDLO–HPMC(P) extruded, (**b**) DMF–HPMC(AS) extruded at 5% and 50% API loading.

The PXRD of crystalline NaDLO exhibited peaks at 15.23, 19.96, 25.0, 25.90 and 27.16° at 2θ values. After melt extrusion with 50% *w*/*w* HPMC(P), the characteristic peaks of crystalline NaDLO were present, but the intensity of the peaks was reduced. The 5% *w*/*w* NaDLO solid dispersion with HPMC(P) produced an amorphous halo indicating the solubilization of APIs in polymer melt forming solid solution [57] (Figure 7a). The PXRD of crystalline DMF (Figure 7b) showed characteristic diffraction peaks at 2θ values 11.08°, 17.68°, 22.11°, 24.12°, and 27.5° [58]. The PXRD of 50% *w*/*w* DMF solid dispersion with HPMC(AS) exhibited diffraction peak characteristics of crystalline DMF with reduced intensity, indicating incomplete crystalline–amorphous transformation. The PXRD of pure polymers are shown in Figure S2d. Solid dispersion with HPMC (AS) containing 5% *w*/*w* DMF produced an amorphous halo, confirming the complete amorphization of DMF. In addition to PXRD, the FTIR spectra of NaDLO exhibited distinctive peaks at 3388.57 cm^−1^ due to NH stretching of the secondary amine, at 1576.82 cm^−1^ owing to C=O stretching of the carboxyl ion and at 747.35 cm^−1^ because of C-Cl stretching (Figure S2). In the FTIR of the melt extruded sample of 5% *w*/*w* NaDLO with HPMC(P) containing 5% *w*/*w* NaDLO, a peak at 1576 was still present, showing no interaction between APIs and the polymer (Figure S2a) [59]. The FTIR spectra of crystalline DMF showed 1672 cm^−1^ for the C=C stretching vibration, 1719 and 3430 cm^−1^ for the C=O stretching vibration, 1160 cm^−1^ for the C−O stretching vibration, and 670–890 cm^−1^ and 2850–3080 cm^−1^ for the C−H bending vibration. These peaks were not found in either the 5% *w*/*w* DMF-HPMC(AS) physical mixture or the melt extruded solid dispersion containing 5% DMF [60] (Figure S2b). For comparison, the FTIR of pure polymers has been shown in Figure S2c.

#### 3.3.3. Dissolution Experiments

**Figure 8:**
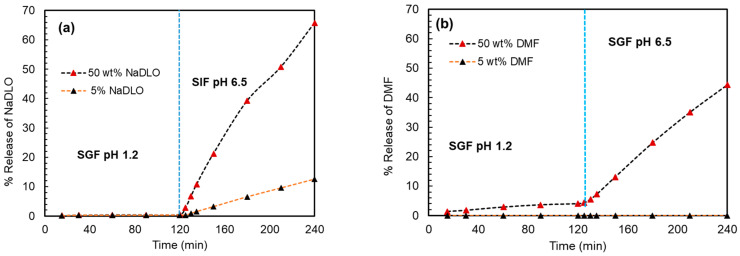
The release of (**a**) NaDLO from extruded samples with HPMC(P), and (**b**) DMF from extruded samples with HPMC(AS) under simulated gastric fluid (SGF) and simulated intestinal fluid (SIF) environments.

The dissolutions of the extruded samples were performed according to the protocol for enteric dosage forms. The solid dispersion of 5% *w*/*w* NaDLO with HPMC(P) showed no release in SGF pH 1.2 for 2 h. When exposed to SIF pH 6.8, HPMC(P) solid dispersion released 10% of the drug. In the case of solid dispersion containing 50% *w*/*w* NaDLO, 65–75% drug release was observed in 2 h (Figure 8a). At a pH below the pKa of the carboxylic acid group in diclofenac (around 4.0), majority of diclofenac sodium will be in the neutral form. As the pH increases above the pKa, the proportion of ionized diclofenac will increase, with a maximum at around pH 4.5 to 5.5. Above this pH range, the proportion of ionized diclofenac will decrease as the carboxylic acid group becomes fully deprotonated and the molecule becomes negatively charged [61]. This could be a possible explanation as to why solid dispersion of 5% *w*/*w* NaDLO with HPMC(P) showed no release in SGF pH 1.2 for 2 h. Solid dispersion of 5% *w*/*w* DMF with HPMC(AS) showed no release in both SGF pH 1.2 and SIF pH 6.8, whereas solid dispersion containing 50% DMF started releasing DMF in SGF pH 1.2 (5% in 2 h) and in SIF pH 6.8, incomplete release (42% in 2 h) was observed (Figure 8b). Although both polymers are soluble in SIF pH 6.8, the negligible dissolution of the drug at 5% *w*/*w* loading could be due to a strong interaction between the APIs and the polymer.

### 3.4. Effect of Drug Loading

**Figure 9:**
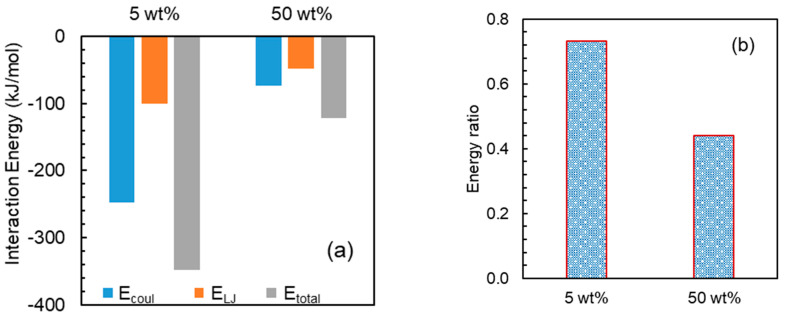
(**a**) Interaction energies between NaDLO and HPMC(P) and (**b**) energy ratio: NaDLO– HPMC(P)/NaDLO–NaDLO at 5 and 50 wt% NaDLO.

**Figure 10:**
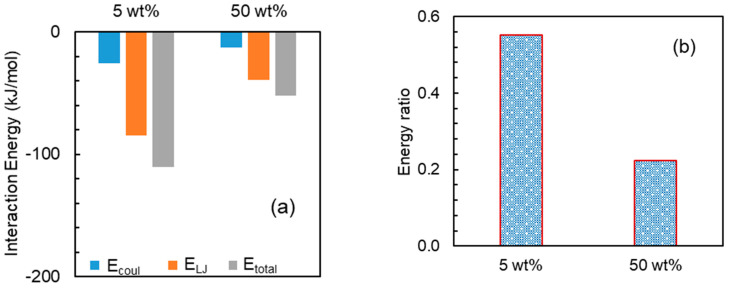
(**a**) Interaction energies between DMF and HPMC(AS) and (**b**) energy ratio: DMF– HPMC(AS)/DMF–DMF at 5 and 50 wt% DMF.

Analysis from experiments reflects that higher loading is suitable for faster API release. To gain insights into this observation at the atomic level, further MD simulations were performed at higher loadings corresponding to the feasible pairs, i.e., NaDLO–HPMC(P) and DMF–HPMC(AS) obtained by experiments. Figure 9a shows the interaction energy between NaDLO and HPMC(P). As the loading of NaDLO in HPMC(P) increases from 5 wt% to 50 wt%, all interaction energy terms E_coul_, E_LJ_, and E_total_ decrease. This indicates that the strength of binding between NaDLO and HPMC(P) is reduced. Due to reduced interaction or weaker binding, NaDLO shows faster release at higher loading, as observed in experiments. Consistent with interaction energies, the energy ratio also indicates weaker relative interaction between NaDLO and HPMC(P) at higher loading, as shown in Figure 9b, which supports faster NaDLO release. It is instructive to observe NaDLO–NaDLO at both loadings. Figure S3a depicts E_coul_, E_LJ_, and E_total_ at both loadings. Similarly to NaDLO–HPMC(P) interactions, all interaction terms in NaDLO–NaDLO decrease as loading increases; however, Na^+^-DLO^−^ interaction increases with loading (Figure S3b). Interestingly, E_LJ_ interactions between Na^+^ and DLO^−^ ions are repulsive, as indicated by positive energy values. Owing to very strong ionic interaction between Na^+^ and DLO^−^, these ions reside very close to each other and hence LJ interactions are repulsive. Figure 10 shows the interaction energies between DMF and HPMC(AS) as well as the energy ratios at both loadings. With the increase in DMF loading from 5 wt% to 50 wt%, both the DMF–HPMC(AS) interaction and the energy ratio are reduced, indicating weaker binding between DMF and HPMC(AS), hence supporting faster DMF release at higher loading, as observed in experiments. Despite a decrease in DMF–HPMC(AS) interaction, DMF–DMF interaction showed a reverse trend with loading (Figure S4) as seen in the case of Na^+^-DLO^−^ interaction.

**Figure 11:**
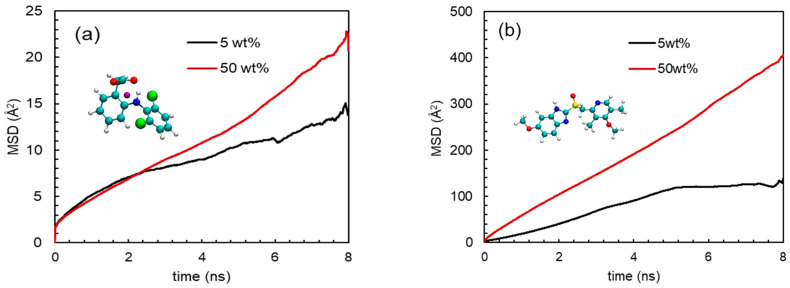
Mean-squared deviations (MSDs) of (**a**) DLO in HPMC(P) and (**b**) DMF in HPMC(AS) at 5 and 50 wt% loadings of each API.

The release phenomena of an API from solid dispersion could efficiently be illustrated by dynamics of the API in solid dispersion. Usually, the dynamics of a molecule in a mixture environment is evaluated by mean-squared displacement (MSD) [62,63]. Not only the dynamics, but also the diffusion modes of a molecule/particle can be characterized by MSD [64]. The MSD is quantified as

(2) $$\mathrm{MSD}(t)=\frac{1}{N}\sum_{i=1}^{N}\langle |r_{i}(t)-r_{i}(0){|}^{2}\rangle$$

where *N* is the number of active molecules and **r***_i_*(*t*) is the position of the *i*th active molecule at time *t*. To be precise, MSD was computed based on the trajectory from 2 ns to 10 ns. Figure 11 depicts the MSDs of NaDLO and DMF in HPMC(P) and HPMC(AS), respectively, at 5 and 50 wt% loadings. As simulation time proceeds, the MSDs continuously increase. As the loading of NaDLO increases from 5 to 50 wt%, the mobility of the NaDLO is enhanced, as indicated by the increased MSD value at 50 wt% (Figure 11a), which, in turn, indicates faster release of NaDLO from solid dispersion. This supports the experimental observation obtained from the release study (Figure 8a). Similar to the MSD of NaDLO in HPMC(P), the MSD of DMF in HPMC(AS) also depicts that, with an increase in loading, the mobility of the DMF is raised (Figure 11b). This is also consistent with experimental observation (Figure 8b). Overall, upon increasing the loading of API, API–polymer interaction decreases, which results in higher mobility of APIs in solid dispersions, thus boosting the release of APIs in the SIF environment.

### 3.5. Effect of Temperature

**Figure 12:**
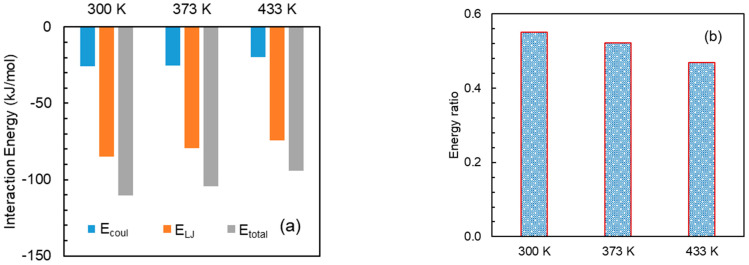
(**a**) Interaction energies between DMF and HPMC(AS), and (**b**) energy ratio: DMF–HPMC(AS)/DMF–DMF at temperatures of 300, 373, and 433 K.

**Figure 13:**
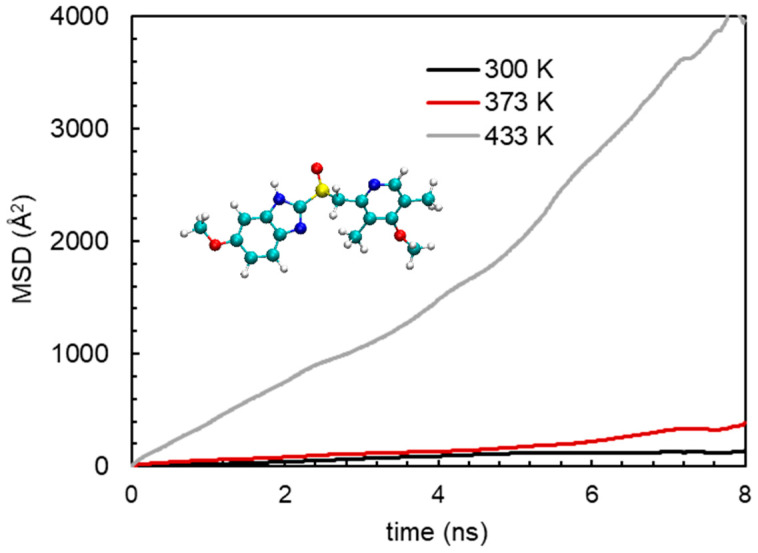
Mean-squared deviations (MSDs) of DMF in HPMC(AS) at temperatures of 300, 373, and 433 K.

To elucidate the effect of temperature at the molecular level, a model system, i.e., a mixture of DMF and HPMC(AS) was considered. Particularly, simulations were performed at three different temperatures at 300 K, 373 K and 433 K, respectively. It should be noted that the examined temperatures are below the degradation temperature of DMF. Figure 12a shows the interaction energies between DMF and HPMC(AS) at various temperatures. The hierarchy of all the DMF–HPMC(AS) interaction terms (E_coul_, E_LJ_, and E_total_) is [DMF–HPMC(AS)]_433 K_ < [DMF-HPMC(AS)]_373 K_ < [DMF-HPMC(AS)]_300 K_. This indicates that DMF would be strongly bounded to HPMC(AS) at a lower temperature. As expected, energy ratios indicate the same hierarchy as the interaction trend (Figure 12b). Furthermore, the dynamics of DMF in the mixture of DMF and HPMC(AS) at these temperatures are illustrated to predict the release behaviour. Figure 13 depicts the MSDs of DMF at 300 K, 373 K, and 433 K, particularly for the last 8 ns simulation. As the temperature increases, the mobility of DMF is enhanced, as indicated by significantly increased MSD values at 433 K compared to 300 K. Weaker DMF–HPMC(AS) interaction at a higher temperature, as explained above, results in higher mobility of DMF, which would support the release of DMF from solid dispersion. In brief, a higher temperature would be expected to boost the API release phenomena.

## 4. Conclusions

To investigate molecular interactions in solid dispersions, MD simulations and experimental approaches were adopted. The interaction energies between NPX and polymer in terms of E_coul_, E_LJ_, and E_total_ increased in the order of HPMC(AS) < HPMC(P) < Eudragit L100, whereas for NaDLO, these quantities increased in the order of Eudragit L100 < HPMC(AS) < HPMC(P). For DMF and OPZ, the hierarchies of the energy terms are Eudragit L100 < HPMC(P) < HPMC(AS) and HPMC(P) < Eudragit L100 < HPMC(AS). The suggested API–polymer pairs are NPX-Eudragit L100, NaDLO-HPMC(P), DMF-HPMC(AS) and OPZ-HPMC(AS), respectively. The energy ratio trends of API–polymer combinations are consistent with the interaction energy hierarchies. All APIs were able to form hydrogen bonds with polymeric excipients. Being ionic in nature, NaDLO showed a higher number of hydrogen bonds compared to the other APIs. Among non-ionic APIs, OPZ had a higher number of hydrogen bonds. The pairs NaDLO–HPMC(P) and DMF–HPMC(AS) were successfully extruded by HME experiments. The release studies revealed that NaDLO and DMF APIs could be loaded up to 50 wt% in HPMC(P) and HPMC(AS), respectively, as these dispersions are rarely released in SGF but are mostly released in SIF. API–polymer interaction decreases as API loading increases, which leads to a higher mobility of API, thus boosting the release of API. The MSD indicates that with an increase in temperature, the mobility of API increases, resulting in a faster release of API. This study, in suggesting a potential polymeric excipient for delayed-release APIs, provides atomic-level insights into the compatibility between APIs and polymeric carriers, which could accelerate the rational design and development of a solid dispersion system for poorly soluble APIs.

## Figures and Tables

**Table 1:** Temperature profile used for HME of various API polymer combinations.

Barrel Zone	1	2	3	4	5	6	Rod Die
Temperature (°C)							
NaDLO-HPMC(P)	Feeding	110	140	140	145	145	145
DMF-HPMC(AS)	Feeding	75	100	110	120	120	115
NPX-Eudragit L100	Feeding	130	200	215	220	220	215
OPZ-HPMC(AS)	Feeding	75	100	110	120	120	115

**Table 2:** Simulated densities and lattice parameters of drug crystals. The experimental values are mentioned in ().

API	Temp (K)	Density (g/cc)	Lattice Parameters						Ref.
			a (Å)	b (Å)	c (Å)	α (°)	β (°)	γ (°)	
Naproxen	300 (283–303)	1.27 (1.25)	13.30 (13.38)	5.76 (5.79)	7.87 (7.91)	90.0 (90.0)	93.9 (93.9)	90.0 (90.0)	[41]
Diclofenac sodium	150 (150)	1.48 (1.44)	9.48 (9.55)	39.19 (39.49)	9.77 (9.84)	90.0 (90.0)	90.7 (90.7)	90.0 (90.0)	[42]
Dimethyl fumarate	150 (150)	1.43 (1.43)	3.87 (3.87)	5.64 (5.64)	8.36 (8.36)	100.8 (100.8)	100.3 (100.3)	105.7 (105.7)	[43]
Omeprazole	300 (283–303)	1.31 (1.33)	9.81 (9.70)	10.49 (10.29)	10.45 (10.62)	90.0 (90.4)	111.5 (112.1)	116.5 (115.9)	[44]

## Data Availability

Literature data that support our results are properly cited in the respective sections.

## Supplementary Materials

The following supporting information can be downloaded at: https://www.mdpi.com/article/10.3390/pharmaceutics15041164/s1, Table S1. Solubilities, melting points, and degradation temperatures of the API and polymers; Table S2: Number of Hydrogen bonds between polymer excipients and APIs; Figure S1: MD simulation snapshots; Figure S2: FTIR spectra of at 5% *w*/*w* API loading in (a) NaDLO–HPMC(P) physical mixture and extruded, (b) DMF–HPMC(AS) physical mixture and extruded, (c) FTIR and (d) PXRD of pure polymers; Figure S3: Interaction energies between: NaDLO and NaDLO, and Na^+^ and DLO at 5 and 50 wt% NaDLO; Figure S4: Interaction energies between DMF and DMF at 5 and 50 wt% DMF.
