# Supplementary material for: Molecular Interactions between APIs and Enteric Polymeric Excipients in Solid Dispersion: Insights from Molecular Simulations and Experiments
Source: Pharmaceutics. 2023 Apr 6;15(4):1164. doi: 10.3390/pharmaceutics15041164 (PMC10143979; doi:10.3390/pharmaceutics15041164)
Supplement: Supplementary file 1 [file pharmaceutics-15-01164-s001.zip › pharmaceutics-2272813-supplementary.pdf]

Molecular Interactions between APIs and Enteric Polymeric Excipients in Solid Dispersion: Insights from Molecular Simulations and Experiments

Krishna M. Gupta[1]*, Xavier Chin[1] and Parijat Kanaujia[1, 2]*

1. Institute of Sustainability for Chemicals, Energy and Environment (ISCE[2]), Agency for Science, Technology and Research (A*STAR), 1 Pesek Road, Jurong Island, Singapore 627833, Republic of Singapore
2. Department of Pharmacy, National University of Singapore, 18 Science Drive 4, Singapore 117559

Table S1. Solubilities, melting points, and degradation temperatures of the API and polymers.

**Drug**	**MW (Da)**	**Solubility**	**Melting point/Tg** (°C)	**Degradation temp (°C)**
Diclofenac Sodium	318.13	Soluble in water	285	285
Naproxen	230.25	Practically insoluble at pH below 4	155	196
Dimethyl fumarate	144.12	Soluble in water	102	Not reported
Omeprazole	345.4	Very slightly soluble in water	156	156
HPMC-P 55	35000	Soluble above pH 5.5	143	199
HPMC-AS (LF)	16000	Soluble above pH 5.5	122	204
Eudragit L100-55	135000	Soluble above pH 5.5	120	170

Table S2. Number of Hydrogen-bonds between polymer excipients and APIs.

**Polymer excipients**	**Drug APIs**			
	**Naproxen**	**Diclofenac sodium**	**Dimethyl fumarate**	**Omeprazole**
HPMC(P)	0.73	1.37	0.36	1.29
HPMC(AS)	0.86	1.84	0.62	1.50
Eudragit L100	1.07	1.61	0.93	1.78

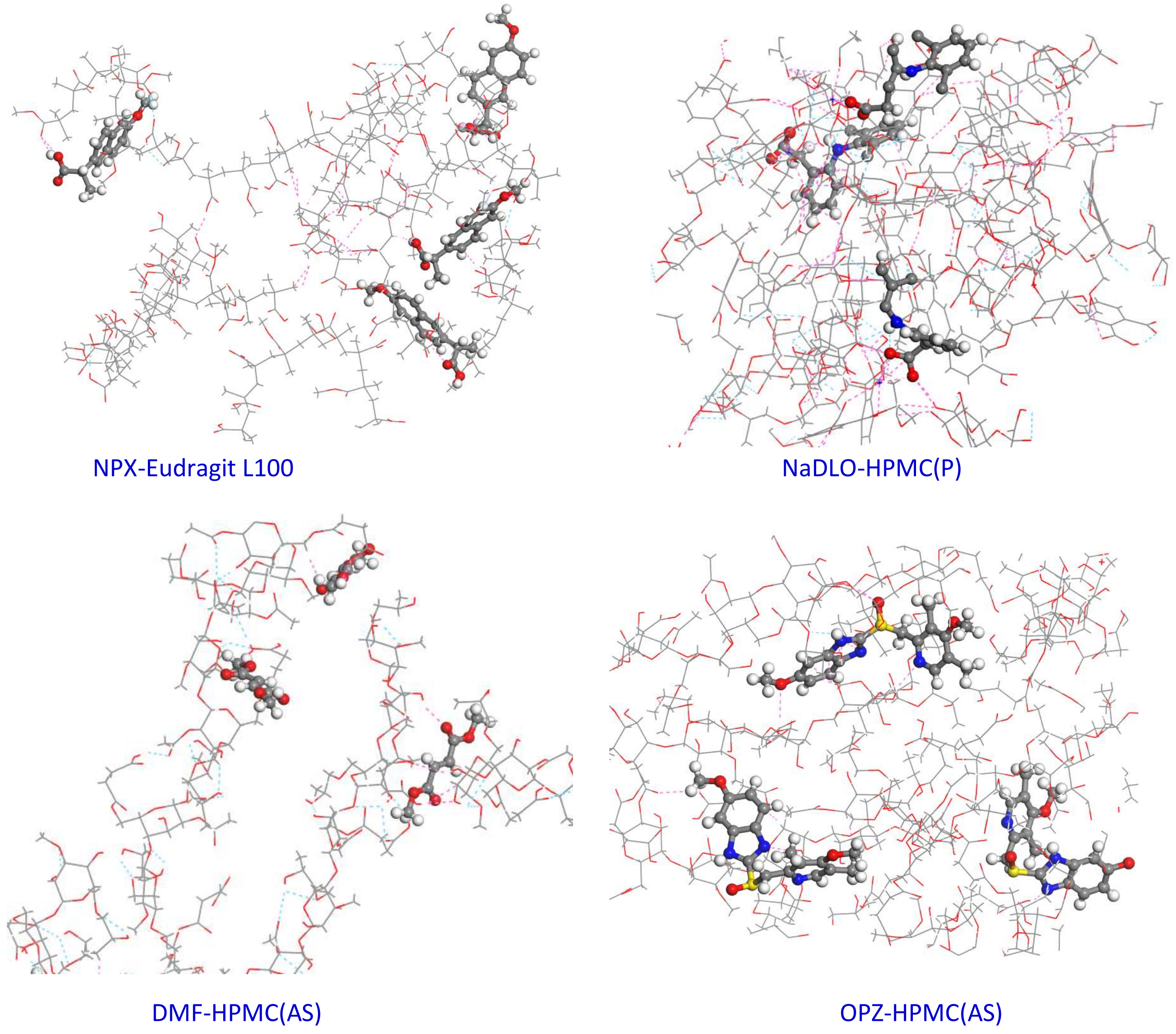

Fig. S1. Part of the final snapshots from MD simulations corresponding to the best API-polymer pairs.

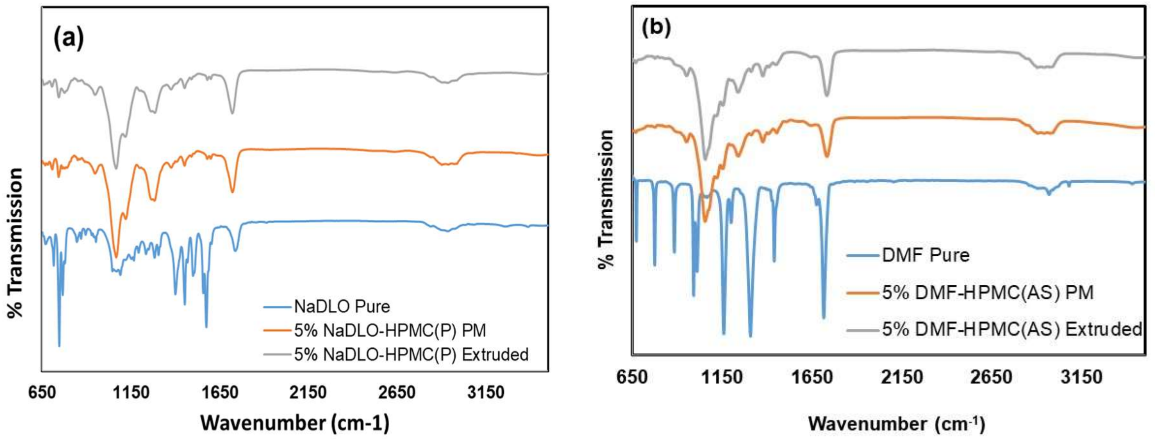

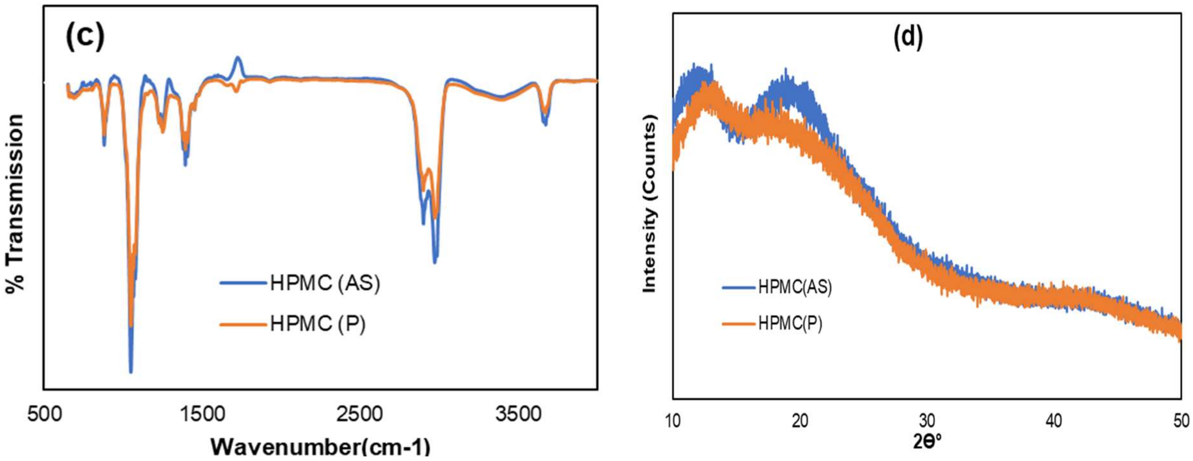

Fig. S2. FTIR spectra of at 5% w/w API loading in (a) NaDLO–HPMC(P) physical mixture and extruded, (b) DMF– HPMC(AS) physical mixture and extruded, (c) FTIR and (d) PXRD of pure polymers.

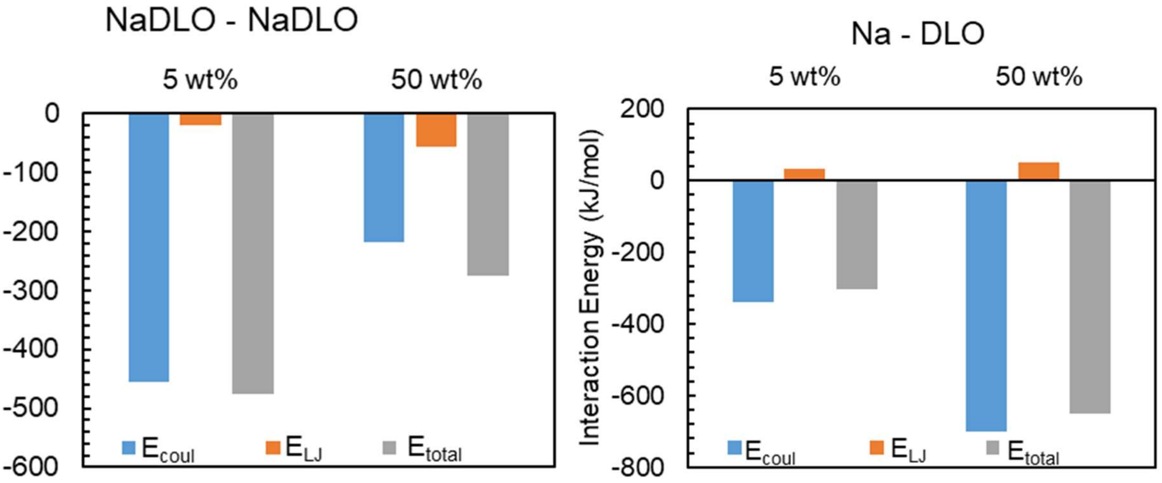

Fig. S3. Interaction energies between: NaDLO and NaDLO, and Na^+ and DLO at 5 and 50 wt% NaDLO.

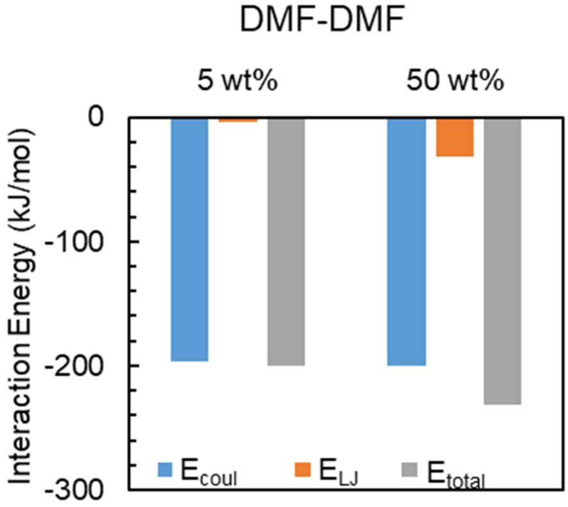

Fig. S4. Interaction energies between DMF and DMF at 5 and 50 wt% DMF.
